# Metabolism-associated molecular classification of cervical cancer

**DOI:** 10.1186/s12905-023-02712-6

**Published:** 2023-10-26

**Authors:** Min Zhao, Xue Zhang, Qing Huan, Meng Dong

**Affiliations:** 1 School of Medicine, Mianyang Central Hospital, University of Electronic Science and Technology of China, Mianyang, 621000 Sichuan China; 2 School of Life Sciences, China Medical University, Shenyang, China; 3 Shandong Key Laboratory of Reproductive Medicine, Department of Obstetrics and Gynecology, Shandong Provincial Hospital, Shandong First Medical University, Jinan, 250021 Shandong China

**Keywords:** Cervical squamous cell carcinoma and endocervical adenocarcinoma, Metabolism, Weighted gene co-expression network analysis, Prognosis, Genetic abnormality

## Abstract

**Objective:**

This study aimed to explore metabolic abnormalities in cervical squamous cell carcinoma and endocervical adenocarcinoma (CESC) for metabolism-related genes.

**Methods:**

We downloaded expression data for metabolism-related genes, performed differential expression analysis, and applied weighted gene co-expression network analysis (WGCNA) to identify metabolism-related functional modules. We obtained normalised miRNA expression data and identified master methylation regulators for metabolism-related genes. Cox regression of data on metabolism-related genes was performed to screen for genes that affect the prognosis of patients with CESC. Furthermore, we selected key genes for validation.

**Results:**

Our results identified 3620 metabolism-related genes in CESC, 2493 of which contained related mutations. The co-occurrence of *CUBN*, *KALRN*, and *HERC1* was related to the prognosis of CESC. The fraction of genome altered (FGA) closely correlated with overall survival. In expression analysis, 374 genes were related to the occurrence and prognosis of CESC. We then identified four metabolic pathway modules in WGCNA. Further analysis revealed that glycolysis/gluconeogenesis was related to endothelial cells and that arachidonic acid metabolism was related to cell proliferation. These four modules were also related to the prognosis of CESC. Among CESC-related metabolic genes, two genes were found to be regulated by microRNAs (miRNAs) and methylation, whereas another two genes were coregulated by miRNAs and mutations.

**Conclusions:**

Among metabolism-related genes, 15 genes were related to the prognosis of CESC. The co-occurrence of *CUBN/KALRN/HERC1* was associated with CESC prognosis. Glycolysis/gluconeogenesis was related to endothelial cells, and arachidonic acid metabolism was related to cell proliferation.

**Supplementary Information:**

The online version contains supplementary material available at 10.1186/s12905-023-02712-6.

## Introduction

Cervical cancer carries a high risk of morbidity and mortality in women in developing countries [1]. Uncontrolled local tumour progression and distant spread of metastases mainly contribute to death of patients [2]. Therefore, it is necessary to elucidate the molecular mechanisms of CESC.

Metabolic activities have substantial effects on cancer development, progression, and prognosis in various cancers [3], including CESC [4, 5]. Metabolic reprogramming is a common hallmark of tumours [6]. Changes in cell metabolism may promote transformation and tumour progression [7]. Therefore, elucidation of the mechanisms mediating cancer metabolism may have implications in the exploration of basic cancer pathophysiology.

The associations of genetic and epigenetic alterations with metabolic processes in CESC are not fully understood. Increased mutation rates are often observed in human cancers, leading to loss of function (i.e., the mutation renders the gene completely inactive) caused by somatic mutations, copy number variations, and genomic rearrangements. In addition to genetic alterations, gene expression can be modulated by epigenetic mechanisms, such as DNA methylation, histone modification, and noncoding RNA regulation [8]. Online public databases and high-throughput sequencing technology have provided us with platforms for comprehensive genome-wide gene expression analyses to improve our understanding of the impact of genetic composition on various diseases [9].

In this study, we aimed to identify metabolism-related genes in CESC and explore the associations between genomic changes and clinical parameters, including overall survival. Furthermore, we evaluated the differential expression of metabolism-related genes in CESC and used weighted gene co-expression network analysis (WGCNA) to construct a co-expression network of those and screen for significant functional modules. Finally, we explored the mechanisms mediating cancer progression with regard to metabolism-related genes. Overall, our study may provide insights into the molecular classification of patients with CESC.

## Materials and methods

### Data collection

Metabolism-related genes were obtained from the Fluxer database (https://fluxer.umbc.edu/) [10]. RNA sequencing, copy number variants, mutation variants, methylation, microRNA (miRNA) expression data, and clinical data for patients with CESC in The Cancer Genome Atlas (TCGA) datasets were downloaded from UCSC XENA (https://xena.ucsc.edu/).

### Somatic mutation and copy number analyses

Among the mutation types, we evaluated frame shift insertions and deletions, nonsense mutations, nonstop mutations, slice site mutations, and translation start site mutations as mutations related to loss of function. The effects of these genes on prognosis in patients with CESC were observed using Kaplan–Meier log-rank tests. In order to further elucidate the effects of metabolism-related gene mutations in patients with CESC, we used the above gene mutation information to calculate the tumour mutation burden (TMB) and the fraction of genome altered (FGA) for each sample.

### RNA-sequencing analysis

For comparisons of tumour samples with normal tissues, we performed differential expression analysis using the ‘Deseq2’ package. Differentially expressed genes were defined as having a false discovery rate less than 0.05 and |log2(fold change)| greater than 1.

#### WGCNA

In order to elucidate the roles of metabolism-related genes in CESC, we selected genes related to the occurrence and prognosis of CESC using WGCNA [11]. A gene co-expression network was constructed using the ‘WGCNA’ package in R [11].

### miRNA expression analysis

To study the mechanisms underlying dysregulated metabolism-related genes in cancer, we identified master miRNA regulators for metabolism-related genes based on two criteria. Cytoscape was used to visualise the network of miRNAs and metabolism-related genes.

### DNA methylation analysis

For the methylation probe, probes in the promoter region were selected. We identified master methylation regulators for metabolism-related genes according to the Pearson correlation of methylation with the expression of the target gene (*P* < 0.05 and R^2^ < -0.3).

### Construction of metabolism-related signature of CESC

We first performed single-factor Cox regression with data on metabolism-related genes to screen for genes that affect the prognosis of patients with CESC. Furthermore, because of the collinearity between genes, we used LASSO to screen independent genes. A metabolism-related signature was constructed by combining clinical parameters of CESC-related cases. The ‘Glmnet’ package was used to perform LASSO analysis.

### Cell culture

HeLa cell line was purchased from cell bank of the Chinese Scientific Academy and was cultured in Dulbecco’s modified Eagle medium (Gibco, Life Technologies, 11,965,092, Grand Island, NY, USA) with 10% foetal bovine serum (Gibco, Life Technologies, 1,981,614, Grand Island, NY, USA) and 1% penicillin–streptomycin at 37 °C in a humidified incubator containing 5% CO_2_.

### Small interfering RNA (siRNA) and reverse transcription quantitative polymerase chain reaction (RT-qPCR)

Specific siRNAs (Sangon Biotech, Shanghai, China) were transfected into HeLa cells using Lipofectamine2000 (Invitrogen, 2,067,429, Carlsbad, CA, USA). Forty-eight hours later, the cells were harvested for further experiments. An RNA simple Total RNA Kit (QIAGEN, DP430, Beijing, China) was used to extract RNA from the cell lines, and RNA was reverse transcribed into cDNA using a Reverse Transcription Kit (Takara, RR047Q, Tokyo, Japan). SYBR Green (Takara, 640,022, Tokyo, Japan) was used for qPCR analysis on a Roche LightCycler 96 real-time PCR system. Details of the components of the reaction solution and the procedures used for PCR are described in Supplementary Tables 1 and 2. The results were normalised to the expression of glyceraldehyde-3-phosphate dehydrogenase (*GAPDH*), and the comparative CT (2^−ΔΔCT^) method was used to determine the expression levels of *CUBN*, *HERC1*, and *KALRN* relative to *GAPDH*.

### Western blotting

Total protein from HeLa cells was extracted using cell lysis buffer. Western blotting was performed by separating proteins on 6–12% polyacrylamide gels, transferring proteins to polyvinylidene difluoride membranes, and immunodetection of specific proteins using enhanced chemiluminescence. Proteins were visualised using an imaging system. Antibodies targeting KALRN (19,740–1-AP) and β-actin (20,536–1-AP, 1:5000) were obtained from Proteintech (China).

### Immunofluorescence

HeLa cells were inoculated on coverslips and processed. After transfection of siRNA for 48 h, cells were fixed with 4% paraformaldehyde for 10 min at room temperature. All slides were then washed with PBS and blocked with goat serum for 30 min at room temperature. Cells were incubated with primary antibody (KALRN [Proteintech, China], 1:1000) overnight at 4 °C, washed with PBS, and then incubated with the appropriate secondary antibody for 1 h at room temperature. Cells were immediately examined with Revolve Generation 2 fluorescence microscope.

### Cell viability

The HeLa cells with or without transfection were plated in 96-well plates (3 × 10^4^ cells per well, with 200 μL culture medium) and cultured at 37 °C in a humidified incubator containing 5% CO_2_. Cell viability was evaluated using a Cell Counting Kit-8 (MedChemExpress, HY-K0301, Shanghai, China) at 1–5 days following the manufacturer’s instructions. The absorbance value was measured at 450 nm after 1 h of incubation at 37 °C in an incubator containing 5% CO_2_.

### Statistical analysis

R V4.1.0 was used for data analysis. We used the ‘maftools’ package to analyse gene mutations and copy numbers. The ‘survival’ package was used to perform prognostic analysis. We used t-tests to compare differences between two groups if they were normally distributed and Wilcoxon tests if they did not conform to a normal distribution. If there were more than two groups, analysis of variance was used to compare differences between groups if they were normally distributed, and Kruskal–Wallis tests were used if the data were not normally distributed. Pearson correlation analysis was used to compare correlations between genes and pathways. Results with *P*-values less than 0.05 were considered significant.

### Ethics Statement

No animals or humans were included in this study, therefore no statement for ethical permission is needed.

## Results

### Genomic changes in metabolism-related genes in CESC

**Fig. 1:**
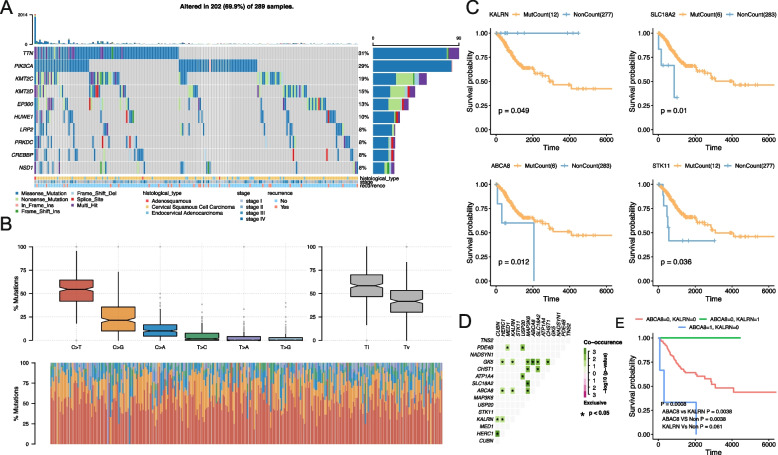
Mutation status of metabolism-related genes in cervical cancer. **A** Mutation types of the top 10 most frequently mutated genes in cervical cancer. **B** Nucleotide changes in metabolism-related genes. **C** Mutation-related genes that affect prognosis (partial). **D** Co-occurrence analysis of gene mutations related to the prognosis of cervical cancer. **E** Prognostic analysis of combined mutations in the *CUBN*, *KALRN*, and *HERC1* genes

From the Fluxer database, we compiled a total of 3620 genes related to metabolism in CESC; these genes were regarded as metabolism-related genes for subsequent analyses. Additionally, 2493 of these genes harboured CESC-related mutations. As shown in Fig. 1A, the top three genes with the highest mutation frequency were *TTN* (31% mutation rate), *PIK3CA* (29% mutation rate), and *KMT2C* (19% mutation rate). Moreover, we found that nucleotide mutation of C > T was the most frequent among all mutation types in CESC (Fig. 1B).

In order to determine whether loss-of-function mutations affected the prognosis of patients with CESC, we analysed the number of mutations in more than five samples, this analysis yielded 616 genes. From this prognostic analysis, loss-of-function mutations in 15 genes, including *KALRN*, *SLC18A2*, *ABCA8*, *STK11*, *ATP1A4*, *CHST1*, *CUBN*, *GK5*, *HERC1*, *MAP3K6*, *MED1*, *NADSYN1*, *PDE4B*, T*NS2*, and *USP20* were found to be closely related to prognosis in patients with CESC (Supplementary Table 1 and Fig. 1C). Owing to the mutual influence between gene mutations, we further explored mutually exclusive/co-occurrence associations among these 15 prognosis-related genes. After analysis, we found that there was no mutual exclusion between prognostic-related gene mutations, although mutual occurrences between the eight pairs of genes were detected (Fig. 1D). Therefore, we conducted a joint prognostic analysis of these eight gene pairs. After analysis, we found that the three gene pairs *KALRN/CUBN*, *HERC1/CUBN*, and *KALRN/HERC1* were related to the prognosis of CESC (Supplementary Table 2).

**Fig. 2:**
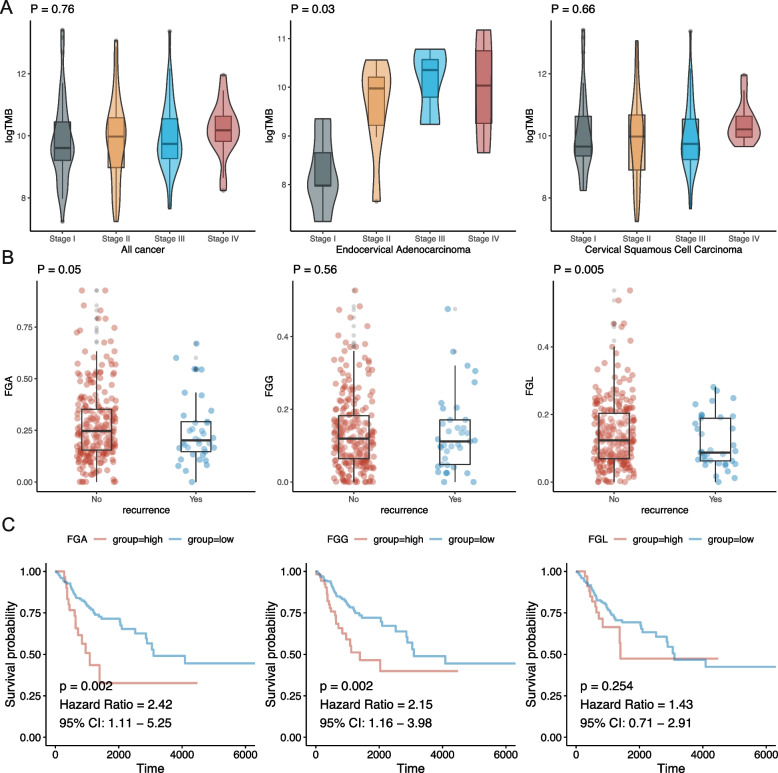
Genomic characteristics of metabolism-related genes in cervical cancer. **A** Relationships between tumour staging and tumour mutation burden (TMB). **B** Association between metabolism-related fragment changes and histological type. **C** Association between metabolism-related fragment changes and tumour prognosis

To further assess the relationships between overall mutations in metabolism-related genes and CESC, we calculated the TMB score based on all mutations in metabolism-related genes. We evaluated the associations of TMB scores with clinical stage, histological type, recurrence, and prognosis. The results showed that correlations of prognosis and clinical parameters of CESC with TMB were not meaningful (Supplementary Table 3). However, in an analysis based on histological grouping (excluding adenosquamous samples owing to small sample size), we found that clinical stage was associated with metabolism-related TMB in endocervical adenocarcinoma (Fig. 2A and Supplementary Table 4).

In addition to genomic mutations, we also observed variations in copy numbers. Thus, we calculated the FGA, fraction of genome gain, and fraction of genome loss (FGL) based on changes in the copy numbers of metabolism-related genes. After analysis, we found that FGL has differences among different histological types (Fig. 2B and Supplementary Table 5). In addition, in the prognostic analysis, it was found that FGA and FGG were related to the prognosis of CESC (Fig. 2C).

### Expression and functions of metabolism-related genes in CESC

**Fig. 3:**
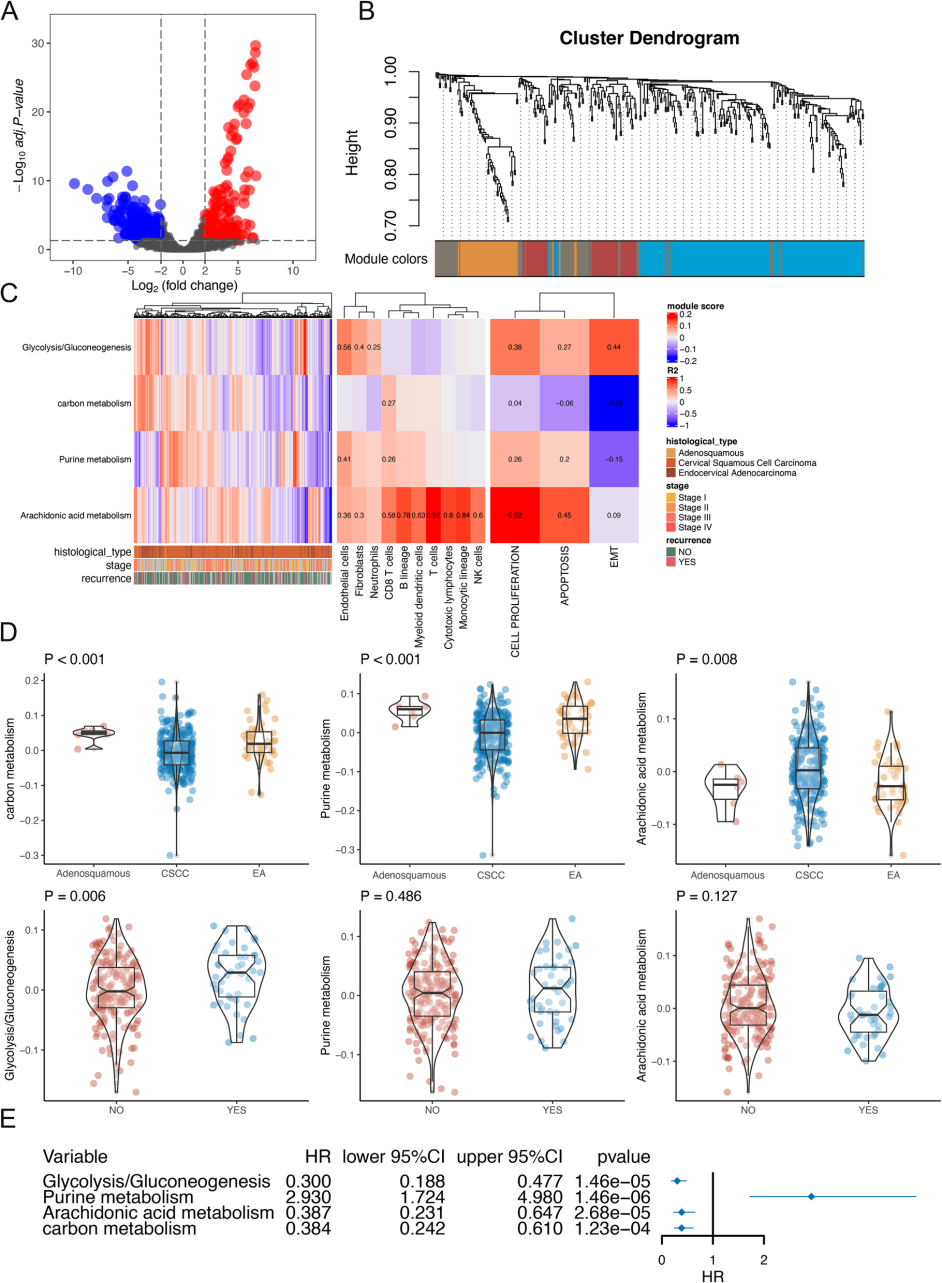
Metabolism-related gene functional analysis. **A** Differential expression of metabolism-related genes in cervical cancer. **B** Module grouping of metabolism-related genes. **C** Relationship between metabolism-related modules and tumour immunity and related phenotypes. **D** Relationship between metabolism-related modules and clinical features. **E** Association between metabolism-related modules and prognosis in patients with cervical cancer

In total, 234 upregulated and 260 downregulated genes in CESC were identified by differential expression analysis of metabolism-related genes (Fig. 3A and Supplementary Table 6). Additional analyses showed that 374 of these genes were significant for the prognosis of patients with CESC (Supplementary Table 7). In order to elucidate the biological functions of these genes in CESC, we performed molecular clustering analysis using the WGCNA algorithm to construct a co-expression network. After evaluation of the preliminary data, we chose five as the candidate threshold for co-expression network construction (Fig. 3A). By fitting genes with similar expression levels into a module, we obtained four modules, which contained 55–133 genes each (Fig. 3B). We performed functional annotations on the four modules through enrichment analysis and finally found that these four modules were related to arachidonic acid metabolism, carbon metabolism, glycolysis/gluconeogenesis, and purine metabolism. Therefore, the corresponding metabolic pathways were used to represent each module (Supplementary Table 8).

Next, we applied the MCP counter algorithm to evaluate the immune infiltration of each sample and to improve our understanding of the correlations between these modules and tumour-related immune cells. The results showed that the arachidonic acid metabolism module was related to most immune cells and that glycolysis/gluconeogenesis was related to endothelial cells (Fig. 3C). Additionally, using the GSVA algorithm, we evaluated the scores of tumour-related phenotypes (cell proliferation, apoptosis, and the epithelial-mesenchymal transition) in patients with CESC. The results showed that the arachidonic acid metabolism module was related to cell proliferation (Fig. 3C).

The histological classification was mainly associated with carbon metabolism, purine metabolism, and arachidonic acid metabolism modules. Moreover, the correlation between tumour recurrence and the glycolysis/gluconeogenesis module was also significant (Fig. 3D). Finally, we conducted a survival analysis for each metabolism module and showed that each metabolism-related module was related to the prognosis of CESC (Fig. 3E).

### Possible regulatory mechanisms of metabolism-related genes

**Fig. 4:**
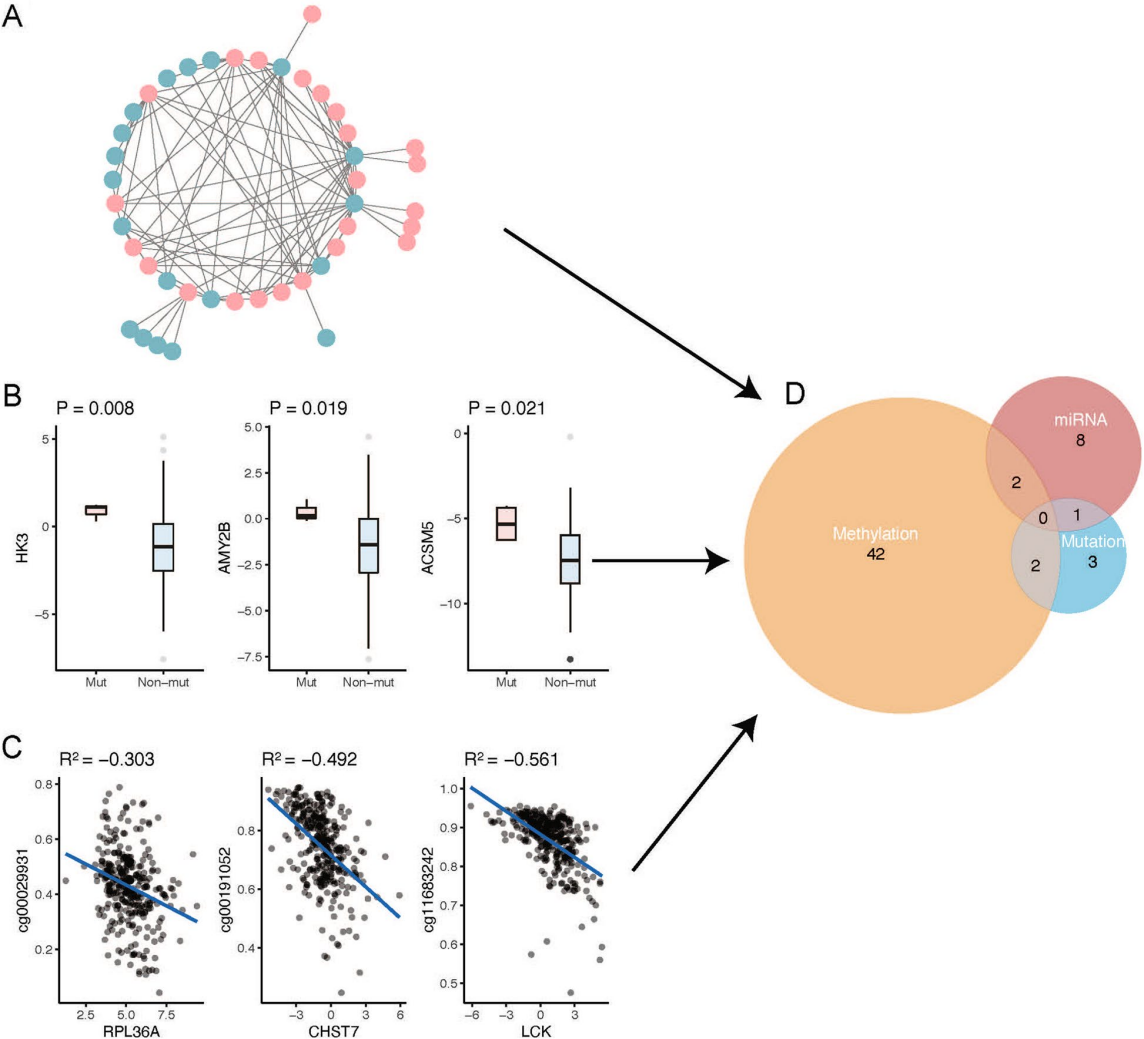
Potential regulatory mechanism of metabolism-related genes in cervical cancer. **A** Metabolism-related genes regulated by miRNA. In the figure, red represents metabolism-related genes, and blue represents miRNA. **B** The top three metabolism-related genes affected by mutation. **C** The top three genes affected by methylation. **D** Venn diagram of comprehensive regulation of metabolism-related genes by three regulatory methods

Through TCGA database analysis, we first analysed the negative regulatory effects of miRNAs on differentially expressed genes. The results showed that there were 11 genes negatively regulated by miRNAs (Fig. 4A, Supplementary Table 9). Furthermore, we evaluated the effects of mutations on gene expression and showed that the expression levels of six genes were affected by their mutations (Fig. 4B, Supplementary Table 9). Analysis of the effects of promoter methylation on gene expression revealed that 46 genes may be affected by methylation (Fig. 4C, Supplementary Table 9). Thus, 58 genes were regulated by the above three mechanisms. Importantly, *NME5* and *SLC40A1* were affected by methylation and miRNA regulation, whereas *HK3* and *JAK3* were regulated by both mutations and methylation (Fig. 4D).

### Construction of a cell metabolism-related signature

**Fig. 5:**
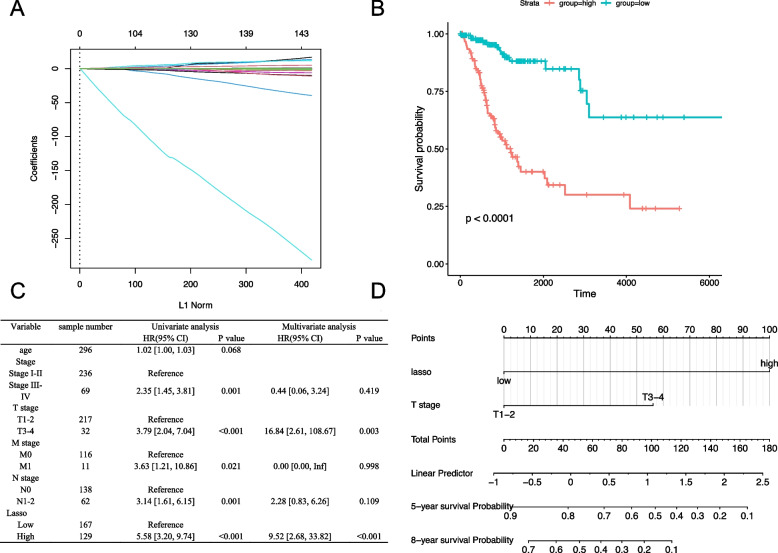
Construction of CESC-related metabolism model. **A** Lasso regression analysis selects the variables included in the model. The dotted line in the figure represents the lambda value. Each line represents a gene. The genes to the left of the dotted line belong to the meaningful genes. **B** Prognostic analysis of metabolism-related models. **C** Prognostic analysis of CESC-related clinical features. **D** The nomogram of CESC-related features. Each variable can correspond to a score above. Each patient calculates a score based on her status

We performed Cox regression to identify the genes that affect the survival of patients with CESC using LASSO to screen out genes with collinearity (Fig. 5A). After analysis, we found that the lambda value of LASSO was 0.0759. Finally, candidate genes were selected based on this lambda value; 14 genes affected the prognosis of patients with CESC. Furthermore, we constructed a metabolism-related signature using these 14 genes. The prognostic analysis of the signature reminded us that the high expression group was closely correlated with worse survival (Fig. 5B). Finally, multifactor COX regression analysis was used to evaluate the cell metabolism-related signature and clinical parameters of CESC. We found that the cell metabolism-related signature and T stage were the most significant factors affecting prognosis in patients with CESC (Fig. 5C). Therefore, based on the above two variables, a nomogram was constructed to indicate the contribution of each variable to the outcome and to predict the survival probability more directly (Fig. 5D). As shown in the nomogram, high expression of a metabolism-related signature and higher T stage could lead to a poor prognosis.

### Validation of key genes in the CESC

**Fig. 6:**
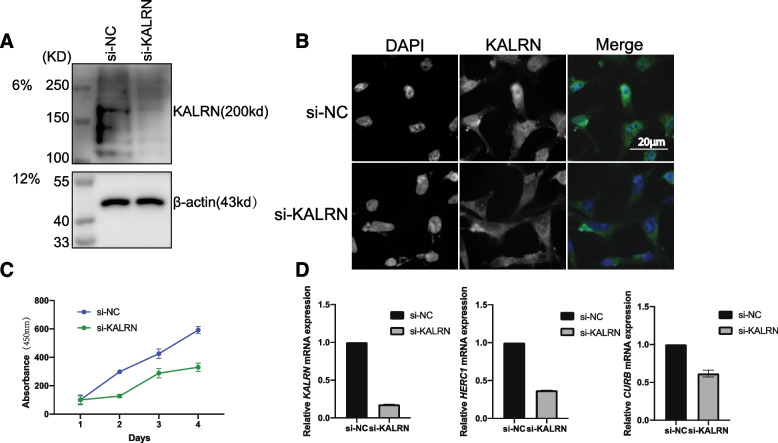
Validation of key genes in the CESC. **A** Knockdown decreased the expression level of KALRN protein. Total protein extracted from Hela cell treated with an siRNA against KALRN (si-KALRN) or with a negative control siRNA(si-NC) for 3 days, analyzed by western blotting with antibodies against the indicated proteins (KALRN and β-actin). **B** The fluorescence intensity of knockdown KALRN decreased. The cells were transfected with siRNA or with a negative control si-NC. After 48 h, the cells were collected for immunofluorescence to detect the change of fluorescence intensity and protein localization. Green indicates KALRN, blue indicates DAPI staining, indicating nucleus; Merge is the overlying of two colors. The scale bar 20 μm. **C** CCK-8 assays were conducted to examine Hela cell viability after the knockdown of KALRN. **D** RT-qPCR was used to detect the expression of KALRN, HERC1 and CURB

To further improve the credibility of our findings, we selected three key genes (*CUBN, KALRN* and *HERC1*) obtained from the screen to test our hypothesis. We performed knockdown experiments on the KALRN protein, which showed a significant decrease in KALRN protein and fluorescence intensity (Fig. 6A, B). In addition, further CCK8 experiments were performed, showing that HeLa cells with si-KALRN exhibited a lower proliferative capacity (Fig. 6C). We also examined the transcript levels of CUBN and HERC1 after the KALRN protein was knocked down (Fig. 6D). Transcript levels of HERC1 were significantly altered in KALRN. Taken together, these results suggest that these key genes play a role in the development of cervical cancer.

## Discussion

In the current study, we analysed the characteristics of metabolism-related genes in cervical cancer, assessed the roles of these genes in carcinogenesis, further exploring their genetic and epigenetic alterations. Our results provide potential directions for studying the overall metabolism of cervical cancer.

Our study found that among metabolism-related genes, *TTN*, *PIK3CA*, and *KMT2C* showed the highest mutation frequencies, and C > T was the most common nucleotide mutation in CESC. The *PIK3CA* gene has been reported to be frequently mutated in patients with cervical cancer in the United States of America [12]. Mutations in PIK3CA affect glucose metabolism and proliferation in cervical cancer by activating the AKT/glycogen synthase kinase 3β/β-catenin signalling pathway [13], thereby reversing resistance to radiotherapy [14]. However, a systematic review indicated that the effects of *PIK3CA* mutations on survival outcomes are unclear, and the majority of the included studies support a the potentially negative effect, primarily among those with squamous cell carcinoma [15]. Similarly, *KMT2C* mutations have been shown to be dominant suppressor gene alterations in cervical cancer [16]. In a recent study, mutations in *PIK3CA*, *KMT2C*, and *TTN* were also found [17], consistent with our findings.

In the subsequent gene mutual exclusion/co-occurrence analyses, we found that *CUBN/KALRN/HERC1*showed a co-occurrence relationship and affected the prognosis of patients with CESC. One study showed that *KALRN* is mutated in some cases of human papilloma virus infection concomitant with oropharyngeal squamous cell carcinoma [18]. However, further studies are needed to evaluate the roles of *HERC1* in CESC.

TMB is the total number of mutations present in a tumour specimen and is an emerging biomarker for response to immunotherapy [19]. We found no significant correlations of TMB with clinical parameters (including overall survival) [20], consistent with a previous report. However, we found that clinical stage could be associated with metabolism-related TMB in CESC.

We also observed changes in copy numbers [21]. Importantly, evaluation of changes in copy numbers of metabolism-related genes showed that FGA, FGG, and FGL were related to recurrence and overall survival in patients with CESC. Similarly, Peng et al. found that *EBP50* played important roles in cell proliferation and that copy number variations in *EBP50* could affect the prognosis of patients with cervical cancer [22].

WGCNA is an unsupervised classification method that can establish connections among co-expressed genes and facilitate the grouping of genes into modules [11]. In this study, we identified four metabolism-related modules of CESC according to WGCNA and pathway enrichment analysis, and each of these modules was verified to be significantly related to prognosis. The arachidonic acid pathway is a metabolic process involved in cervical carcinogenesis by targeting multiple pathways [23], including the cyclooxygenase (COX)-2 pathway; frequent COX-2 expression is closely related to poor prognosis in patients with cervical cancer [24]. Moreover, the metabolic process of glycolysis has been shown to correlate with worse prognosis in patients with cervical cancer [25, 26].

Previous studies have shown that fibroblasts are related to glycolysis/gluconeogenesis [27]. In our study, we also found a positive correlation between fibroblasts and glycolysis/gluconeogenesis (R^2^ = 0.4). In addition, as a metabolic pathway closely related to the occurrence of tumours, arachidonic acid metabolism positively related to most tumour immune cells, with a particularly strong correlation with T cells. Our research was consistent with a previous study, which also confirmed the relationship between arachidonic acid and T cells [28]. Moreover, after further exploring the correlations between these metabolism-related modules and clinical parameters, we speculated that the modules of carbon metabolism, purine metabolism, and arachidonic acid metabolism may be related to different histological classifications and could affect the overall survival of patients with CESC. Glycolysis/gluconeogenesis can influence tumour recurrence and has been shown as an independent prognostic factor for tumour recurrence in patients with advanced CESC treated with definitive chemoradiotherapy [29]. Furthermore, we also discovered that the arachidonic acid metabolism module was related to cell proliferation using the GSVA algorithm, which may attenuate cell proliferation via a mechanism independent of calcium entry, thereby contributing to poor prognosis [30].

We found that 11 genes were negatively regulated by miRNAs and that the expression levels of six genes were affected by their mutations. Additionally, the expression of 46 genes may be affected by promoter methylation. Interestingly, we identified 58 genes that were regulated by the above three mechanisms. *NME5* and *SLC40A1* were affected by methylation and miRNA regulation, whereas *HK3* and *JAK3* were regulated by both mutation and methylation. Promoter methylation in the *SLC40A1* gene has been reported to affect differential expression [31], and the expression of *HK3* has been shown to be upregulated in CpG island methylator phenotype-high tumours [32], supporting our findings. Our findings provided the evidence for further studying the association between genetic variation and expression of differentially expressed genes in CESC.

## Conclusion

In summary, in this study, we identified genomic alterations in metabolism-related genes in patients with CESC and evaluated their expression levels and biological functional modules. The co-occurrence of *CUBN/KALRN/HERC1* was related to the prognosis of patients with CESC. Glycolysis/gluconeogenesis was related to endothelial cells, and arachidonic acid metabolism was related to cell proliferation. We explored the potential mechanisms regulating metabolism-related genes. Our findings provided insights into the metabolic activities in patients with CESC and established potential biomarkers for the carcinogenesis and prognosis of cervical cancer.

### Supplementary Information

**Additional file 1: Figure S1.**

**Additional file 2: Tables S1-S9.**

## Data Availability

The datasets used and analyzed during the current study are available from the corresponding author on reasonable request.
