# Supplementary figures and images for: Metabolism-associated molecular classification of cervical cancer
Source: BMC Womens Health. 2023 Oct 26;23:555. doi: 10.1186/s12905-023-02712-6 (PMC10605340; doi:10.1186/s12905-023-02712-6)

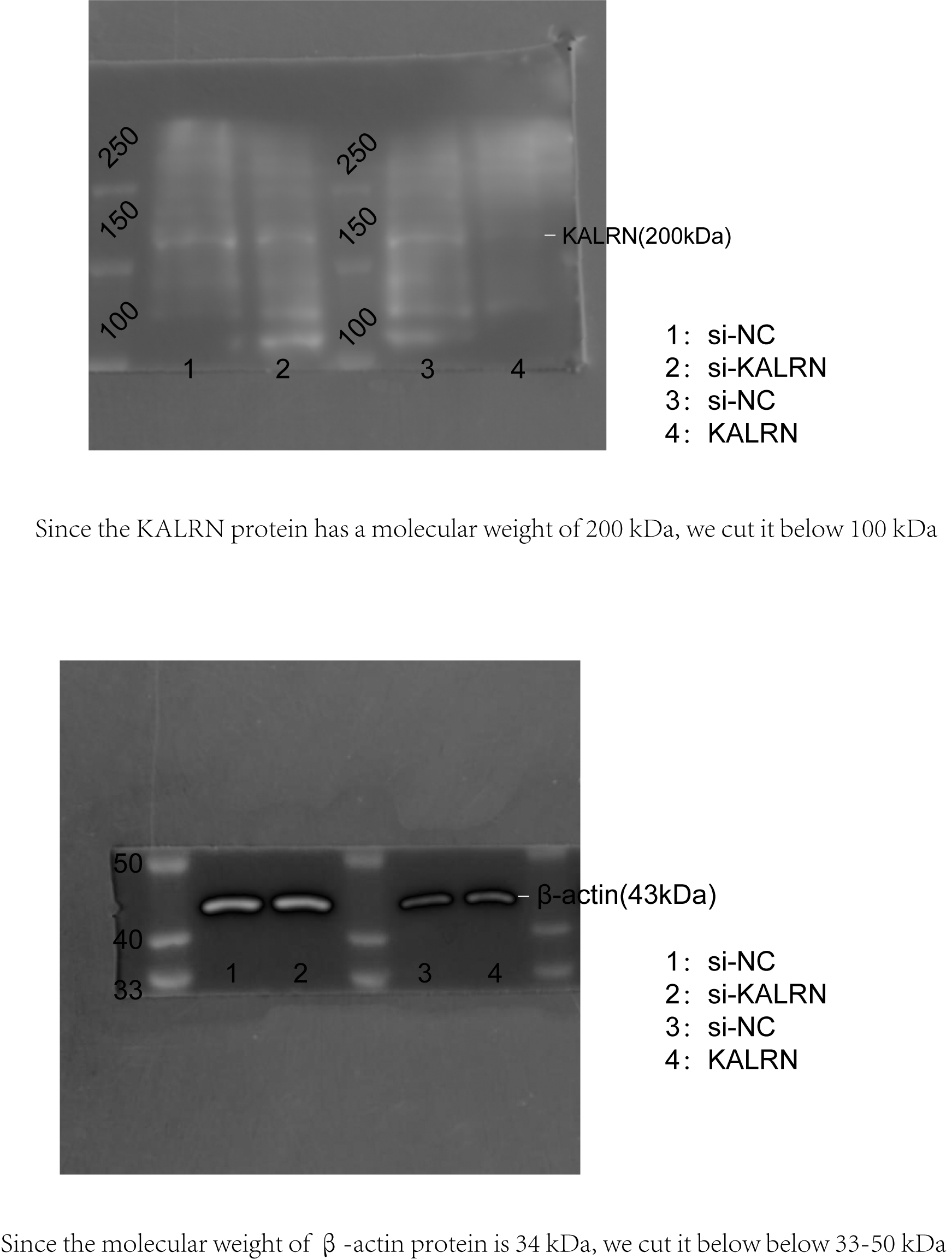

Supplement: Supplementary file 1 — Additional file 1: Figure S1. [file 12905_2023_2712_MOESM1_ESM.jpg]
